# Real-Time Current Volume Estimation System from an Azure Kinect Camera in Pediatric Intensive Care: Technical Development

**DOI:** 10.3390/s25103069

**Published:** 2025-05-13

**Authors:** Florian Chavernac, Kévin Albert, Hoang Vu Huy, Srinivasan Ramachandran, Rita Noumeir, Philippe Jouvet

**Affiliations:** 1 Department of Electrical Engineering, Ecole de Technologie Supérieure (ETS), Montréal, QC H3C 1K3, Canada; florian.chavernac.1@ens.etsmtl.ca (F.C.); hoang.vu-huy.1@ens.etsmtl.ca (H.V.H.); rita.noumeir@etsmtl.ca (R.N.); 2 Department of Pediatrics, Université de Montréal (UdeM), Montréal, QC H3T 1C5, Canada; kevin.albert.hsj@ssss.gouv.qc.ca; 3 Department of Computer Science, University of Petroleum and Energy Studies (UPES), Dehradun 248007, India; srinivasan.ramachandtan@ddn.upes.ac.in; 4 CHU Sainte-Justine Research Centre, Montréal, QC H3T 1C5, Canada

**Keywords:** depth sensor, intensive care, respiratory monitoring, spirometry, automatic, real-time

## Abstract

Monitoring respiratory parameters is essential in pediatric intensive care units (PICUs), yet bedside tidal volume (Vt) measurement is rarely performed due to the need for invasive airflow sensors. We present a real-time, non-contact respiratory monitoring system using the Azure Kinect DK (Microsoft, Redmond, WA, USA) depth camera, specifically designed for use in the PICU. The system automatically tracks thoracic volume variations to derive a comprehensive set of ventilator equivalent parameters: tidal volume, respiratory rate, minute ventilation, inspiratory/expiratory times, I:E ratio, and peak flows. Results are displayed via an ergonomic web interface for clinical use. This system introduces several innovations: real-time estimation of a complete set of respiratory parameters, a novel infrared-based region-of-interest detection method using YOLO-OBBs, enabling robust operation regardless of lighting conditions, even in total darkness, making it ideal for continuous monitoring of sleeping patients, and a pixel-wise 3D volume computation method that achieves a mean absolute error under 5% on tidal volume. The system was evaluated on both a healthy adult (compared to spirometry) and a critically ill child (compared to ventilator data). To our knowledge, this is the first study to validate such a contactless respiratory monitoring system on a non-intubated child in the PICU. Further clinical validation is ongoing.

## 1. Introduction

### 1.1. Motivation

In intensive care units (ICUs), patients have critical conditions that require careful medical care involving monitoring of vital signs to prevent possible dangerous situations [1]. These patients are admitted to the unit after surgery or when they have multiple organ damage and/or severe respiratory infection [2].

In PICUs, the main cause of hospitalization before the age of one year is bronchiolitis, representing a major challenge [3]. It is characterized by an increase in respiratory frequency, signs of respiratory distress, and the presence of crepitating rales, symptoms which may progress to respiratory depression [3]. According to data from the Centre Hospitalier Universitaire Sainte-Justine (CHUSJ), 60% of the 1000 children admitted to intensive care each year show signs of respiratory depression, a condition defined by reduced respiratory frequency and amplitude, often accompanied by snoring or periods of apnea, with a notable drop in oxygen saturation.

Because of their high metabolic rate, children consume more oxygen, requiring increased cardiac output and ventilation. Before the age of 8 years, their gas exchange surface area is reduced and lung dead space is greater, exposing them to rapid deoxygenation in the event of insufficient ventilation [4,5,6]. Early detection of low lung volumes is therefore crucial for prompt initiation of non-invasive ventilation or intubation [7,8]. In this context, monitoring, measurement and optimal management of respiratory function are crucial. There are two categories of patients: those on ventilators, whose respiratory function is closely monitored, and those on spontaneous ventilation, for whom assessment of respiratory function remains complex [9]. “Although experienced healthcare professionals can obtain accurate spirometry on children aged five years and upwards, the ability to perform consistently is from age eight onwards” [10].

This increases the importance of having a system capable of monitoring the respiratory function of non-ventilated patients in real time, to anticipate deterioration and avoid delayed intubation, which could lead to severe complications or even be life-threatening.

The aim of this article is to propose a non-invasive monitoring system based on the use of a depth camera and an integrated infrared imaging system. The system is designed to observe thoracic movements during breathing [11]. The estimation method is based on the following assumption, which has been adopted by a number of existing studies [12,13,14,15]: when the patient is lying motionless in bed and there is no occlusion on the surface of the torso, changes in respiratory volume can be reflected by deformation of the thorax and abdomen. This system needs to be precise and mobile, which will enable it to be used directly at the patient’s bedside, facilitating its adoption in the clinical environment.

Ultimately, this innovative system could not only improve monitoring of respiratory function in pediatric intensive care but also reduce hospitalization time for patients and offer a non-invasive spirometry testing method, thus contributing to reliable and rapid management of children in respiratory distress.

### 1.2. Current State of the Research Field

A variety of techniques have been proposed in the literature for assessing chest wall motion. These approaches can be classified into two broad categories: contact methods (such as magnetometers and respiratory inductive plethysmography) and non-contact methods (such as inductive plethysmography) [16,17,18]. The focus here is on the second category, particularly those using RGB-D cameras such as the Intel® RealSense™ D435I (manufactured by Intel Corporation, Aloha, OR, USA; Hillsboro, OR, USA).

Depth cameras have revolutionized computer vision for respiratory assessment. They can be used to extract various respiratory parameters in real time or post-processed. As illustrated in Table 1, some studies measure basic signals, such as respiratory motion or the respiratory airflow waveforms. Others calculate respiratory rate (RR) or tidal volume (Vt).

In addition to respiratory motion and volume estimation, imaging systems, particularly RGB and RGB-D cameras, have also been used to extract other physiological signals such as heart rate and oxygen saturation. Recent works have demonstrated the feasibility of remote photoplethysmography (rPPG) using RGB images [37,38], while hybrid systems using RGB-D data have enabled multi-parameter monitoring in neonatal intensive care settings [36]. Several studies have evaluated the accuracy of respiratory volume estimation using image-based techniques. For instance, in [36] the proposed method was evaluated on a cohort of three neonates, yielding a mean absolute error (MAE) of 12.81% for tidal volume estimation. In contrast, the study presented in [20] tested the approach on a larger cohort of 44 intensive care unit (ICU) patients, reporting a tidal volume error of −0.5 ± 8.1%.

Both methods rely on extracting a one-dimensional signal from the acquired image data by spatially averaging the pixels within the region of interest (ROI). The resulting volume–time curve is derived by estimating the volume of the ROI over time, using the following relationship:

$$V\left(k\right)=D\left(k\right)\times S$$

where *D*(*k*) denotes the average depth variation for the *k*th image frame, and *S* represents the surface area of the ROI.

### 1.3. Summary of Contributions

We have developed a complete, real-time, contactless respiratory monitoring system capable of estimating a full set of ventilator-equivalent parameters: respiratory rate, tidal volume, minute ventilation, inspiratory and expiratory times, inspiratory-to-expiratory ratio, and peak inspiratory and expiratory flows. The system captures depth video using the Azure Kinect camera and automatically processes the data to generate live respiratory signals and parameter estimates, all displayed through an ergonomic web interface designed for ease of use by medical staff, without requiring prior training.

This work introduces several key innovations. First, the system performs real-time estimation of multiple respiratory parameters, going beyond the limited metrics often found in previous studies. Second, it includes a novel region-of-interest detection method based on YOLO-OBBs trained on infrared images, allowing robust and reliable detection of the thorax regardless of ambient lighting, an essential feature for continuous monitoring in intensive care units, including during nighttime. Third, the system employs a pixel-wise 3D volume estimation algorithm, enabling precise tidal volume measurement with a mean absolute error below 5%. Finally, this study presents the first clinical validation of such a system on a non-intubated, critically ill child in the PICU, confirming its clinical feasibility and practical relevance.

## 2. Materials and Methods

The system consists of an RGB-D camera and a laptop for real-time data acquisition and processing. In terms of camera selection, the Kinect Azure (Microsoft, Redmond, WA, USA) appears to be an appropriate choice over its competitors such as stereoscopy and structured light for the study of respiration. This preference is due to its affordability, comprehensive documentation, availability of a Software Development Kit (SDK) and compatibility with Windows 11 software. In addition, its widespread adoption in the scientific community guarantees a substantial knowledge base and support for the development of our system. Acquisition and interaction with camera data is carried out using the open-source Azure Kinect SDK 1.4.1 and NuGet packages K4AdotNet 1.4.17 [39].

**Figure 1:**
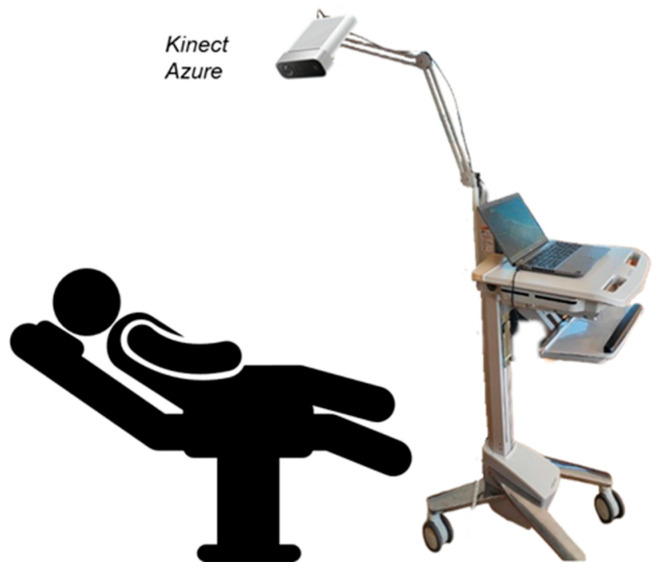
Illustration of the proposed respiratory monitoring system.

The accuracy of the camera’s depth sensor varies according to the distance to the object, over a range from 0.50 m to 3.86 m [40]. At a distance of one meter, the Kinect Azure has an average error of 1.1 mm. [40]. However, the closer you are, the smaller the error is. The resolution of the Kinect Azure’s depth camera depends on the mode selected. For our study, the narrow field of view (NFOV) unbinned (640 × 576) mode [41] was used. In this mode, no pixel binning is applied, meaning that each pixel is preserved without averaging neighboring pixels. This ensures maximum detail in depth measurements. Additionally, the narrow field of view of 75° horizontally and 65° vertically is enough to observe patients. The device is placed arbitrarily above the bed, as long as the camera’s field of view covers the area where the subjects will position themselves, from head to toe. There is no strict requirement for a fixed distance. However, the closer the camera is, the higher the resolution is in the region of interest, which improves measurement precision. This flexibility is possible because the camera is mounted on a mobile stand with an articulated arm, allowing for easy positioning, as illustrated in Figure 1. The camera is generally oriented with an angle close to 90° to remain approximately perpendicular to the patient’s torso. No correction is required for off-center camera position, as the method relies solely on temporal variations in depth. Each pixel’s area contribution is calculated independently, making the system robust to moderate off-center positioning. For replication purposes, we noted afterward that the camera was positioned at a height of 1.1 m above the bed for adults and at 80 cm for the child. Given the hypothesis made, a single camera placed perpendicular to the plane of this movement to ensure that the depth changes captured correspond to vertical displacements was used to measure the breathing movement. The study protocol was approved by the CHUSJ ethics committee (number 2024-6457 & number 2016-1242).

The methodology can be broken down into 4 main parts: detection of the region of interest, calculation of the area and volume within the ROI, calculation of respiratory parameters, and real-time communication between components.

### 2.1. Detection of the ROI

To detect the region of interest (thorax) using the camera, there are several methods with different advantages. The Kinect camera has a body tracking algorithm, which locates the position of the person by estimating the 3D coordinates of key points such as the head, chest, hands, and knees. However, it does not work in many situations, such as lying in bed or in the presence of occlusions such as those encountered in intensive care (ventilation masks, bedsheets, vital sensors, etc.). Moreover, this solution is very costly in terms of computer resources and is not customizable. It is therefore essential to choose a system that is fast, accurate, customizable, and open source. Artificial intelligence algorithms are the best way to meet these requirements for thorax detection. Among them, you only look once (YOLO) [42] stands out for its increased precision and faster execution times. In addition, it runs on GPU-less devices and is easy to install and train.

YOLO offers to perform various tasks with their models, such as object detection, segmentation, image classification, pose estimation, or oriented bounding boxes (OBBs). In our experiments, the YOLO11n-OBB model was selected, as it is the fastest and lightest. In this system, the precise location of the joints is not necessary, as only the position of the oriented box is required to perform the calculations. An oriented bounding box is a rectangular box that can rotate to fit the orientation of the object. In fact, since the objective is to analyze volume variations in space rather than the total volume occupied, it is possible to select an area that includes part of the bed. As long as these remain motionless, they will not introduce additional volume variations.

We needed to train the model so that it could function in the intensive care environment. Two solutions are available for this. The first, more traditional one, is to perform ROI detection on a color image. The second is to use the infrared image captured by the Azure Kinect camera. To make the best choice, we compared the performance of one model trained on color images and another on infrared images. To do this, a dataset was collected consisting of 36 color and infrared (grayscale) images with 640 × 576 px resolution. The images were taken under intensive care conditions at CHUSJ and included data from six healthy adult participants and from 6 pediatric patients hospitalized in the PICU, different from those used for the evaluation of the tidal volume estimation system. For each adult, five images were captured, both clothed and with their torso exposed. No medical equipment (such as electrodes or leads) occluded the torso region of interest in these images. However, for the pediatric patients, standard monitoring equipment may have been present, reflecting typical clinical conditions. To avoid over-fitting, random image processing, including variations in tint, saturation, brightness, scaling, flipping, combining, deleting, and cropping, was applied by YOLO, promoting artificial data augmentation and enriching the diversity of training data [43].

Table 2 presents a comparison of the model’s performance on RGB and infrared (IR) images based on three key metrics: mAP50-95, accuracy, and prediction speed. The results indicate a clear advantage of infrared imaging in terms of both detection performance and processing efficiency. The results of mAP50-95 suggest that infrared data provides more distinguishable features, allowing the model to detect objects with greater reliability. Similarly, the accuracy of the model follows the same trend. Moreover, the system can be used without constraints of ambient lighting conditions [44]. This is an advantage in the PICU environment, where illumination levels vary depending on the time of day or when the patient is sleeping. In addition to its robustness to lighting variation, the use of infrared (IR) images instead of RGB offers a significant privacy advantage. IR imagery conveys fewer facial features and identifiable visual details than RGB, reducing the risk of patient identification. This makes IR particularly suitable for clinical environments such as PICUs, where continuous video monitoring must comply with strict privacy and ethical standards.

**Figure 2:**
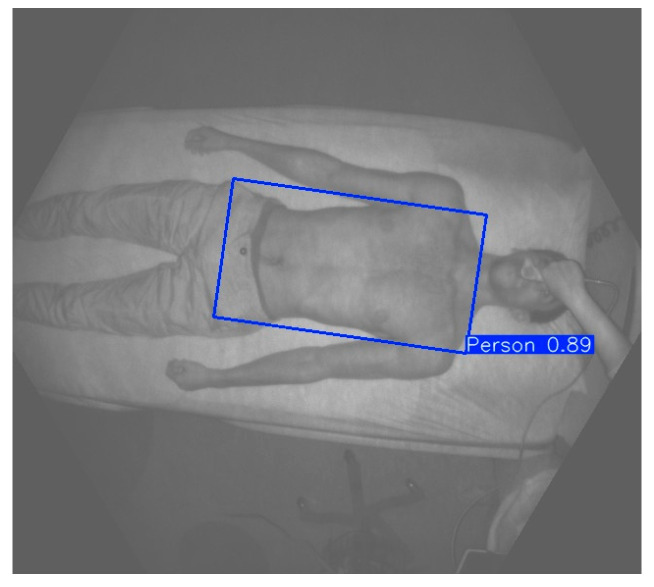
Model prediction developed on a CHUSJ acquisition. The blue rectangle represents the OBB predicted by the model. The number represents the confidence score given by the algorithm.

The trained model is able to make correct predictions even when the human body does not appear entirely in the camera’s field of view, or when several people are present in front of the camera. In this case, the algorithm chooses to observe the area on which the prediction task has given the highest confidence score. The confidence score in the case of an OBB inference represents the model’s certainty about the presence and orientation of the object within the predicted bounding box. A higher confidence score indicates that the model is more certain about the object’s position, shape, and orientation. Figure 2 shows an example of an IR image with a blue rectangle representing the predicted OBB. The confidence score is also displayed.

### 2.2. Area and Volume

Retrieving depth data measured by the Kinect Azure camera is a step made easier using the SDK. However, knowledge of how to use these depth measurements to calculate the volume variation is still needed. The idea is to multiply the depth variation (in mm) by the surface area of the region of interest (in mm^2^) on each image to obtain the volume variation (in mm^3^ converted to mL) between the camera and the region of interest.

Since the system needed to be fully autonomous, it must be able to determine this area without requiring direct measurements on the patient. To achieve this, one solution was to use the tools provided by the Kinect SDK, which enabled transformations between different coordinate systems. Indeed, the color and depth cameras are associated with an independent 2D coordinate system and are also associated with a 3D coordinate system. Taking this information into account, two methods can be used to calculate the area of the region of interest.

The simplest method was to calculate the area considering the four corners, then multiply by the average depth in the zone. However, there were several problems with this method. The first is the effect of distortion caused by the lens placed in front of the sensor [40]. However, the SDK provides a calibration matrix to reduce this distortion effect, inherent to the pinhole camera model. The second problem occurred because not all pixels are at the same sensor depth, creating barrel distortion. This phenomenon means that each pixel does not have an identical area in 3D.

The other method was the one used in the system, as it had the potential to provide a more accurate estimate of volume. This method involves calculating the area of each pixel contained in the ROI. The idea here is that, to estimate the area of a pixel in a depth map, we use the pixel’s immediate neighbors and transform them into 3D using Kinect SDK. By observing the difference in position between these pixels, we estimate the surface area of each of them. The system detects and calculates the ROI area in a single frame for the entire acquisition. This results in a signal that is less affected by variations in surface area.

For each frame captured by the sensor, the depth of the pixel measured is multiplied by its associated area. Each individual volume is summed up to obtain the total volume between the thorax and the camera at a given time t.

### 2.3. Calculation of Respiratory Parameters

The aim of the application was to perform non-invasive spirometry and thus to calculate a set of respiratory parameters. All these parameters were calculated from the change in volume measured by the camera. A zero setting based on the first value was performed, as the aim was to analyze the volume variation rather than the static volume between the camera and the patient’s thorax. The signal was filtered using a finite impulse response (FIR) filter to remove high-frequency noise unrelated to respiratory motion, such as frame-specific pixel fluctuations. The filter cut-off frequency was defined based on known maximum respiratory rate values reported in physiological studies and reference tables [45]. Specifically, an order of 10 and a cut-off frequency of 2 Hz (corresponding to 120 breaths per minute) were chosen. This value ensures attenuation of frequencies higher than the maximum expected respiratory rate and accommodates both pediatric and adult subjects, as children can exhibit significantly higher respiratory rates than adults. These parameters were selected to preserve the morphology of the respiratory signal while attenuating artifacts introduced by system noise. The filtering step also facilitates robust peak and trough detection, which is essential for accurate computation of respiratory cycles. Moreover, it ensures that the derived flow signal is smooth and physiologically coherent, enabling reliable estimation of peak inspiratory and expiratory flow. Before processing the signal, the volume variation must be inverted, as it evolves in the opposite direction to reality. As the volume in the lungs increases, the volume between the camera and the thorax decreases.

A function from the SciPy library (scipy.signal.find_peaks) [46] was used to identify peaks and troughs in the respiratory signal. This function detects local maxima by comparing neighboring samples and allows fine-tuning via parameters such as peak height, prominence, and minimum distance between peaks. Based on the known maximum respiratory rate (RR), we set a minimum distance of 15 frames between consecutive peaks. This corresponds to a maximum RR of 120 breaths per minute (i.e., one peak every 0.5 s), which, at 30 FPS, means a minimum of 15 frames. This setting helps to avoid the detection of multiple successive peaks due to small variations in volume.

**Figure 3:**
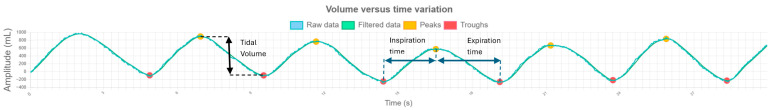
Diagram showing the location of respiratory parameters on the curve of volume variation versus time.

From these markers, respiratory frequency was determined by dividing the number of complete cycles by the total duration measured, from the start of the first cycle to the end of the last. It defines the speed of breathing. The tidal volume of each cycle was obtained by calculating the difference between the peak volume and the corresponding trough volume. Next, the expired volume per minute was calculated by summing all tidal volumes, then dividing by the acquisition time, normalized to one minute. Tidal volume is the amount of air exhaled during each respiratory cycle. Inspiratory time and expiratory time correspond respectively to the duration of inspiration and expiration during a respiratory cycle. Another respiratory parameter displayed by the ventilator is the inspiratory to expiratory (I:E) ratio, which is expressed according to the convention of setting the inspiratory time at 1. To obtain the value of the relative expiratory time, simply divide the average expiratory time by the average inspiratory time. The I:E ratio indicates the proportions of each respiratory cycle devoted to inspiratory and expiratory phases. These parameters can be seen in Figure 3.

Finally, the last parameter to be calculated was the variation in flow rate as a function of time. Flow rate is defined as the derivative of volume with respect to time. In this way, the peak and trough detection steps were applied again. The peak expiratory flow (PEF) was determined by measuring the difference in flow between a peak and the following trough, while the peak inspiratory flow (PIF) was obtained in the opposite direction. Flow rate provides information on the speed at which the air volume is moving.

### 2.4. Real-Time Communication Between Components

To ensure communication between the application retrieving data from the camera and another application in charge of post-processing, a socket connection has been implemented.

To display the results for users, a web application was developed. This type of application has several advantages, such as accessibility and interactivity. The development of such an application requires the rigorous selection of a framework for the backend and another for the frontend. To ensure consistency with CHUSJ’s other applications and to favor open-source solutions, React.js 18.3.1 [47] was chosen for the frontend. Real-time data display was based on Chart.js 4.4.7 [48], an open-source library renowned for its ability to generate responsive, interactive charts with excellent visual rendering. Furthermore, a screen capture function has been integrated directly from the browser, allowing users to export a PNG image of the current visualization. This feature is intended for documentation purposes, such as archiving visualizations or including snapshots in patient reports or medical records.

For the backend, we chose Flask 3.1.0 [49], an open-source Python 3.9.12 micro-framework. The main reasons for this choice were its lightness and ease of integration. Unlike heavier frameworks such as Django 5.2.1 [50], Flask enabled rapid implementation while offering the flexibility needed to manage real-time data flows.

One of the major challenges of the project was to efficiently transmit the respiratory data captured by the Kinect to the web interface, ensuring a continuous, low-latency flow. To achieve this, a Server-Sent Events (SSEs) [51] flow was implemented. SSEs offers several advantages over alternatives such as WebSockets 15.0.1 [52], including optimized one-way communication, and connections treated as conventional HTTP traffic, which can improve the efficiency of server resourcing.

In addition to the main stream of respiratory volumes, other application programming interface (API) routes can be used to send the image of the detected ROI, as well as parameters such as respiratory rate, minute expiratory volume (MEV), tidal volume, I:E, and peak flow.

The application’s ergonomics were validated in collaboration with doctors, its future users. The interface was directly inspired by those used by ventilators in intensive care. This visual consistency reduces the learning curve and may facilitate adoption of the tool in the hospital environment.

## 3. Results

**Figure 4:**
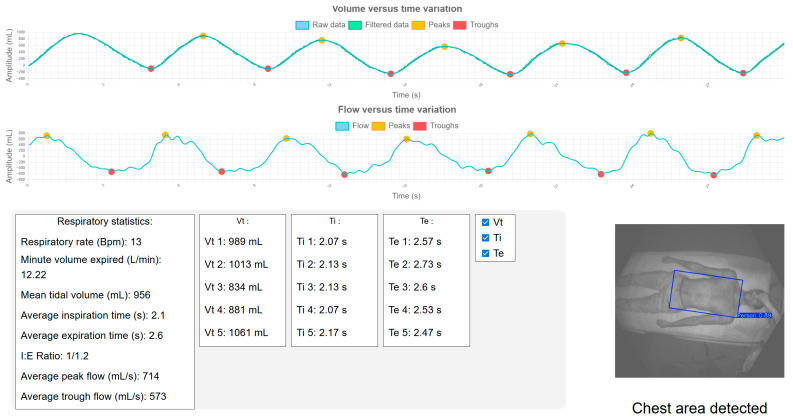
Web interface rendering developed for CHUSJ.

The development of the application enabled us to create a responsive interface, i.e., adaptable to different screen sizes, to keep the placement of all elements visible and coherent. The proposed system is a distributed one, with the emphasis on efficiency and ease of use. A distributed system is ideally suited to the medical environment. The camera can be placed in the room and the results visible from the nurse’s computer without the need to leave their workstation. The clinician can follow all the steps and understand what is going on to check that no inconsistencies occur during execution. In particular, the ROI detection performed is displayed in the web application to visualize the area observed by the algorithm. Several checkboxes are available to access measurements of individual respiratory cycles to verify that no inconsistencies are present. A rendering of the web application is shown in Figure 4. Two sections are displayed: the volume variation graph and the flow graph. These graphs highlight the detected peaks and troughs. Below them, a section presents the calculated respiratory statistics along with the identified ROI.

In terms of computer resource usage. In our tests, carried out on an Intel Core i5-135H processor, the prediction algorithm had an execution time of 179 ms. The processor’s maximum power consumption is 10% of its capacity, and RAM usage is 1.5 GB during calculations.

As a proof of concept, an experiment was carried out to assess the system’s accuracy. The aim was to compare the results obtained by the system with a reference method for volume measurement. To this end, one adult man (23 years old) performed three acquisitions through a spirometer, which is considered the gold standard in the clinical field. Additionally, one critically ill child (one year old) under non-invasive ventilation was included in the study, with the ventilation system serving as the reference. The adult participant, shirtless, laid on a bed in an intensive care room at CHUSJ. He breathed for 30 s through the spirometer, while the camera, positioned approximately 1.10 m above him, recorded the data.

**Figure 5:**
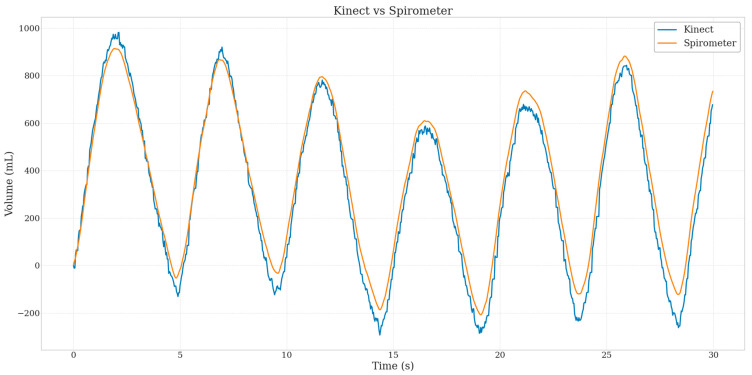
Spirometer and raw Kinect volume–time curves for the adult subject.

**Figure 6:**
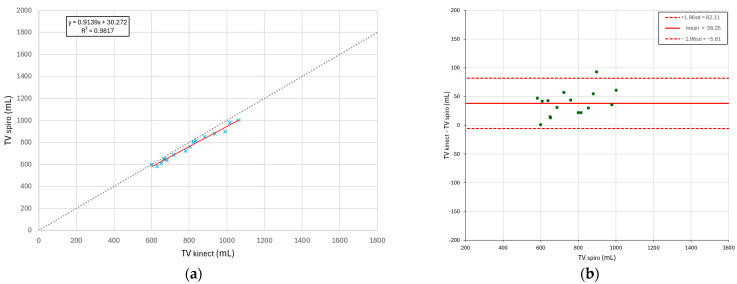
(**a**) Respiratory frequency scatter diagram for the adult subject; (**b**) Bland–Altman diagram for tidal volume for the adult subject.

For the child, the camera was placed at a height of 80 cm. In terms of accuracy assessment, Figure 5 shows that the raw volume signal calculated from the Kinect (blue curve) is highly correlated with the signal from the spirometer (orange curve), with a Pearson correlation index of 0.995 for the adult using the spirometer. For accurate measurement of respiratory parameters for the adult, a scatter diagram for respiratory frequency and a Bland–Altman diagram for tidal volume are shown in Figure 6. In Figure 6a, a correlation coefficient of 0.98 is observed between the measurements provided by our system and those of the spirometer for respiratory rate. In Figure 6b, the central line (solid red line) represents systematic bias, while the two other lines indicate the limits of agreement. Regarding tidal volume, the error remains relatively contained, with a dispersion ranging from 5 to 82 mL.

For the critically ill child, only respiratory rate, expiratory minute volume and tidal volume were available for comparison, as the ventilator provided these parameters. The system demonstrated high accuracy, with an error of 1.5% for the expiratory minute volume, 2% for the tidal volume, and no error for the respiratory rate.

## 4. Discussion

The system demonstrated strong accuracy for this specific healthy adult and critically ill child in intensive care. This study was conceived as a technical proof-of-concept to assess the feasibility of using a Kinect-based system in both controlled and clinical environments. The small sample size was a deliberate design choice to focus on technical validation. A larger ethics-approved clinical study involving a broader pediatric cohort is currently ongoing to evaluate the generalizability, robustness, and clinical performance of the system.

The observed differences between the Kinect-based measurements and the reference spirometer are likely due to the unidirectional nature of depth sensing. Specifically, the system captures thoracic motion only along the optical axis of the camera, potentially missing lateral expansion of the rib cage and other multidirectional breathing movements. This limitation may result in underestimation of respiratory volumes, particularly in patients with more complex breathing mechanics.

In future clinical evaluations within the PICU, additional sources of error are expected, including patient motion and occlusions in the field of view (such as bed sheets, monitoring cables, bandages, or medical devices), which may interfere with accurate signal extraction. Moreover, thoracic morphology and respiratory dynamics vary significantly between infants, children, and adolescents (from 0 to 18 years old), which may affect the reliability of the method across age groups. To address these limitations, improvements such as occlusion-resilient ROI tracking, multi-angle acquisition, and adaptive calibration techniques are planned.

The system’s architecture makes it easy to maintain, as one step, such as ROI detection, can be replaced by another to keep up with advances in the field. Moreover, a similar RGB-D model can replace the camera. Indeed, with Microsoft ending production of the Kinect in August 2023, the system will have to evolve. CHUSJ plans to acquire Orbbec Femto cameras, which are considered clones of the Kinect Azure, developed in partnership with Microsoft. The system will therefore require some minor code modifications to ensure compatibility with these new cameras.

The use of our system to calculate several respiratory parameters is a useful solution for detecting respiratory pathologies such as asthma or chronic obstructive pulmonary diseases (COPDs).

## 5. Conclusions

The results obtained demonstrate that the developed system is optimized for real-time operation while minimizing computational resource requirements. One of the key contributions of this work is the design of an autonomous solution requiring only a Kinect (or Orbbec) camera and a Windows computer. This simplicity in hardware allows for rapid deployment across multiple hospitals, making the solution both accessible and practical for clinical use.

Beyond its accessibility, the system introduces several technical innovations that significantly advance non-contact respiratory monitoring. It performs real-time estimation of a full set of ventilator-level respiratory parameters, enabling continuous bedside assessment. It also includes a novel infrared-based region-of-interest detection using YOLO-OBBs, ensuring robust performance regardless of ambient lighting, which is crucial in intensive care, especially for nighttime monitoring. Furthermore, the system relies on a pixel-wise 3D volume estimation method, which achieves high accuracy in tidal volume measurement (MAE < 5%).

This work presents the first reported clinical validation of a depth camera-based system on a non-intubated, critically ill child in the PICU, confirming its feasibility and clinical potential. In summary, the proposed system represents a significant advancement in real-time, non-invasive respiratory monitoring, paving the way for broader clinical adoption.

## Figures and Tables

**Table 1:** Comparison of measured parameters and their real-time capability in respiratory assessment methods.

Author Name, Year	Measured Parameters	Real-Time
Xia and Siochi, 2012 [19]	Respiratory motion	Yes
L’Her, Nazir, Pateau, and Visvikis, 2022 [20]	RR and Vt	Yes
Addison et al., 2023 [21]	RR	Yes
Seppänen, Kananen, Noponen, Alho, and Seppänen, 2015 [12]	Respiratory airflow waveforms	No
Aoki, Nakamura, Fumoto, Nakahara, and Teraoka, 2015 [22]	TV	No
Martínez and Stiefelhagen, 2012 [23]	RR	Yes
Addison et al., 2022 [24]	RR and Vt	No
Addison, Smit, Jacquel, and Borg, 2020 [25]	RR	No
Yang, Han, andBolic, 2020 [26]	Respiratory airflow waveforms	No
Nakajima, Matsumoto, and Tamura, 2001 [27]	RR	Yes
Bernacchia et al., 2014 [28]	RR	No
Imano et al., 2020 [29]	RR and Vt	No
Yu, Liou, Kuo, Lee, and Hung, 2012 [30]	Respiratory airflow waveforms	Yes
Wijenayake and Park, 2017 [31]	Respiratory airflow waveforms	Yes
Benetazzo, Freddi, Monteriù, and Longhi, 2014 [32]	RR	Yes
Addison, Antunes, Montgomery, Smit, and Borg, 2023 [33]	RR	Yes
Penne, Schaller, Hornegger, and Kuwert, 2008 [34]	Respiratory airflow waveforms	Yes
Rehouma, Noumeir, Masson, Essouri, and Jouvet, 2019 [35]	RR and Vt	Yes
Estévez et al., 2024 [36]	RR, Vt, HR, SpO_2_	Yes

RR: respiratory rate; Vt: tidal volume, HR: heart rate, SpO_2_: oxygen saturation.

**Table 2:** Comparison of different metrics depending on the modality studied (traditional color (RGB) vs. infrared (IR) images).

Metrics	RGB	IR
mAP50-95 (Mean Average Precision)	0.77	0.93
Accuracy	0.7	1
Prediction speed (in ms)	213	179

Accuracy in the ROI segmentation task is defined as the proportion of correctly predicted oriented bounding boxes (OBBs), where a prediction is considered correct if the intersection over union (IoU) with the ground truth is greater than 0.5. Predicted speeds are based on an Intel Core i5-135H processor.

## Data Availability

The database generated during the current study is not publicly available due to institutional restrictions on data sharing and privacy concerns. However, it is accessible for research purposes given the approval from the Research Ethics Board of CHU Sainte-Justine is obtained.

## Abbreviations

The following abbreviations are used in this manuscript:

PICU	pediatric intensive care unit
MAE	mean absolute error
ICU	intensive care unit
CHUSJ	Centre Hospitalier Universitaire Sainte Justine
RR	respiratory rate
Vt	tidal volume
ROI	region of interest
SDK	software development kit
OBBs	oriented bounding boxes
IR	infrared
I:E	inspiratory-to-expiratory ratio
PEF	peak expiratory flow
PIF	peak inspiratory flow
